# Monitoring Methodology for an AI Tool for Breast Cancer Screening Deployed in Clinical Centers

**DOI:** 10.3390/life13020440

**Published:** 2023-02-04

**Authors:** Carlos Aguilar, Serena Pacilè, Nicolas Weber, Pierre Fillard

**Affiliations:** Therapixel, 06200 Nice, France

**Keywords:** artificial intelligence, breast cancer, mammography, monitoring

## Abstract

We propose a methodology for monitoring an artificial intelligence (AI) tool for breast cancer screening when deployed in clinical centers. An AI trained to detect suspicious regions of interest in the four views of a mammogram and to characterize their level of suspicion with a score ranging from one (low suspicion) to ten (high suspicion of malignancy) was deployed in four radiological centers across the US. Results were collected between April 2021 and December 2022, resulting in a dataset of 36,581 AI records. To assess the behavior of the AI, its score distribution in each center was compared to a reference distribution obtained in silico using the Pearson correlation coefficient (PCC) between each center AI score distribution and the reference. The estimated PCCs were 0.998 [min: 0.993, max: 0.999] for center US-1, 0.975 [min: 0.923, max: 0.986] for US-2, 0.995 [min: 0.972, max: 0.998] for US-3 and 0.994 [min: 0.962, max: 0.982] for US-4. These values show that the AI behaved as expected. Low PCC values could be used to trigger an alert, which would facilitate the detection of software malfunctions. This methodology can help create new indicators to improve monitoring of software deployed in hospitals.

## 1. Introduction

Artificial intelligence-based tools (AI) have been present for several years in the clinical setting and are increasingly important in decisions regarding patient’s health status. The role of this new type of tool is particularly relevant in fields that rely on image analysis, such as radiology [1,2,3,4]. In breast imaging, AI has been successfully used to characterize lesions on digital images and is on the road to cost-effectiveness by enhancing early detection of cancers, thus improving patients’ long-term survival [5,6,7]. AI is therefore gradually becoming one of the radiologist’s best advisors. One of the use cases of such tools is determining the level of risk that a patient has of testing positive for the disease (i.e., breast cancer), which may be indicated as a score (the higher the score, the greater the risk of testing positive).

Given the importance of AI’s impact on decisions about a patient’s health status, operation and performance of this tool must be subject to strict monitoring [4]. Consequently, AI must not only be evaluated before being deployed in a clinical setting, but also continuously monitored after its deployment.

Monitoring AI-based software deployed in a clinical setting involves identifying any type of software-related abnormalities (including cyber security problems) before they develop or spread [8]. This can range from developing tools that block viruses or unauthorized users [9], to implementing tools that evaluate the reliability of the decision made by the algorithm [10].

A few studies have proposed introducing statistical tools to help visualize and detect several types of deviations related to AI performance [11]. These studies are based on the detection of deviations by defining limits that the predicted values cannot exceed. When not-to-be-exceeded limits are applied, this kind of tool can be effective enough to detect and discard outliers. In the case of breast cancer detection, it is not possible to apply a not-to-be-exceeded threshold at the level of the scores predicted by the AI, because the ground truth establishment is not temporarily close to the prediction, which makes this approach unsuitable. When ground truth is temporally too distant to be used for evaluation, another approach must be considered: we chose to use retrospective evaluation data as a reference to test whether the AI was behaving as expected.

This paper proposes a methodology for monitoring and automatically detecting unexpected behavior of an AI for breast cancer screening when deployed in clinical settings. The method consisted of comparing the scores predicted by the AI in clinical settings with the scores obtained from a retrospective evaluation.

## 2. Materials and Methods

### 2.1. AI System

The AI system used in this study is trained to detect suspicious regions of interest in the 4 views of a mammogram and to characterize their level of suspicion with a score ranging from 1 (low suspicion) to 10 (high suspicion of malignancy). This system uses two groups of deep convolutional neural networks combined with an aggregation module.

This system has been proven to be effective in improving breast cancer detection [12]. Different versions of the tool are deployed in several clinical centers and analyzed accordingly in the present study.

When the AI analyzes a mammogram, it automatically generates an anonymous report. Each report contains the score assigned to any lesion identified on the image, as well as information concerning the modality, i.e., full-field digital mammography (FFDM) or digital breast tomosynthesis (DBT). A report also contains the version of the algorithm used for the analysis (1.2-US, 1.3-US, 2.01-US).

### 2.2. Data

Reports were anonymously collected from 4 radiological centers across the US between April 2021 and December 2022, resulting in a dataset of 36,581 AI records. Table 1 summarizes the characteristics of the data set extracted for this study. AI versions 1.2 and 1.3 were developed to process FFDM images (2D), while version 2.0.1 was developed to process DBT images (3D). To assess the score distribution of each center, a reference distribution is required.

Data to create each reference come from a retrospective evaluation (RE) conducted for regulatory purposes prior to the deployment of the AI in a clinical setting. The main purpose of the RE is to run the algorithm on an external dataset representative of the target population on which the AI will be deployed. These data have not been used for algorithm development to ascertain its generalizability [8,13,14]. In this study, a reference is a distribution of scores obtained in the RE for a specific version of the AI, manufacturer and modality.

Therefore, a reference represents the behavior expected from the AI and constitutes the control point from which any deviation could indicate a malfunction of the tool.

Some of the characteristics of the dataset used to create the references are summarized in Table 2. In total, 13,433 exams were used to create the reference distribution for the 1.2 AI version, 25,330 exams were used for the 1.3 AI version and 14,187 exams were used for the 2.0 AI version. Each of these distributions is then used for statistical analysis (explained in the next section).

### 2.3. Statistical Analysis

To assess the behavior of the AI, its score distribution in each center was compared to a reference distribution matching the AI version, image modality (i.e., FFDM or DBT) and the manufacturer used by the center (e.g., Hologic, Fuji or GE). Data from each center were analyzed globally (over the entire duration of the data extraction) and locally (over each month).

Similarity between distributions was estimated using the histogram correlation which consists of calculating the Pearson correlation coefficient (PCC) between each center AI score distribution and the reference. The coefficient from a given distribution is computed as explained in the following paragraph.

Let be *p* a histogram representing a data distribution. To construct a histogram from a score distribution (ranging from 1 to 10), we split this feature into 10 consecutive and non-overlapping bins $p_{i}$. In the histogram, each bin represents the number of cases whose score is equal to the bin score. For example, $p_{5}$ represents the number of cases having 5 as score. PCC compares only the corresponding bins of two histograms. To compare the histograms *p* = {$p_{i}$} and *q* = {$q_{i}$}, this technique only measures the difference between the bins that are in the same interval; that is, they only compare bins $p_{i\;}$ and $q_{i.}$ ∀i={1,…,10}. The PCC is obtained with the following Formula (1) (where $\bar{p}\;$and $\bar{q}$ are the histogram means):

(1) $$PCC=\frac{\sum_{1}^{10}(p_{i}-\bar{p\;})\left(q_{i}-\bar{q}\right)}{\sqrt{\sum_{1}^{10}{\left(p_{i}-\bar{p}\right)}^{2}\sum_{1}^{10}{\left(q_{i}-\bar{q}\right)}^{2}}}$$

In order to calculate the PCC corresponding to the comparison between the data of each center and each reference, we directly use this formula with *p* as the center histogram and q the corresponding reference. The class represented by each bin is then each score value predicted by the AI: {1, …, 10}. This formula is a direct adaptation of the Pearson formula for calculating the correlation between two histograms.

The closer the PCC value is to 1, the more similar the distributions. Conversely, the closer this value is to zero, the less association there is between the two distributions [15]. A PCC greater than 0.5 is usually considered strong. Between 0.3 and 0.5, correlation is moderate, and between 0 and 0.3, correlation is weak. It is generally accepted that a very strong correlation corresponds to a value greater than 0.9 [16]. In the context of the present study, the closer a PCC is to 1, the more the AI is behaving as expected.

To test the significance of the difference between the reference and the examined center, a goodness-of-fit test was used. This kind of test represents the measure of the compatibility of a sample with an expected probability distribution [17]. A chi-squared (χ²) test was used to assess the significance (*p*-value) of the difference between each center AI score distribution and the reference at a level of significance (α), also called the alpha-level, equal to 0.05. The null hypothesis is that two histograms represent identical distributions. Any *p*-value below α represents a significant difference between the AI behavior in a center and the reference, so the smaller the *p*-value the less the AI behaves as expected.

When abnormal behavior is detected, it is important to be able to distinguish deviations that are relatively not harmful from those that may have serious consequences. A dangerous deviation is, for example, a decrease in scores above 7 accompanied by an increase in scores below 4, since it could potentially lead to false negatives. In order to catch and quantify these types of deviations, we added the Wasserstein distance (W). This metric is widely applied in statistics and machine learning for its ability to quantify the distance between two distributions as the displacements to be made between the bins to pass from one histogram to another [18].

Statistical calculations were performed in Python 3.8.8 [19]. For the Pearson correlation calculation (PCC) and the chi-squared test, Python bindings for the OpenCV library were used [20]. Wasserstein distance (WD) was estimated using the Python module scipy.stats [21].

## 3. Results

### 3.1. Correlation between Reference and Center

The global PCCs between each center distribution and its related reference were 0.998 (*p* < 0.00001) [min: 0.993, max: 0.999] for center US-1, 0.975 (*p* < 0.00001) [min: 0.923, max: 0.986] for US-2, 0.995 (*p* < 0.00001) [min: 0.972, max: 0.998] for US-3 and 0.994 (*p* < 0.00001) [min: 0.962, max: 0.982] for US-4. Comparing the center US-1 with an unmatched reference (wrong manufacturer: a distribution on mammograms with a Hologic system compared to a reference distribution obtained from an image acquired with the Fuji system) resulted in a PCC of 0.374 (*p* = 0.287), thus showing weak or no correlation. Table 3 displays the averages (and standard deviations) over all the months of the PCC for each of the centers. It is important to note that the PCC mean is calculated from the PCCs per month for each center, and this value is different from an overall PCC, which is calculated from the entire distribution of a center over the entire data set.

**Figure 1:**
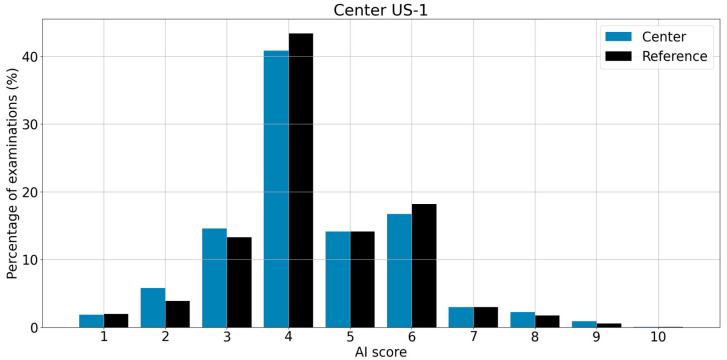
Distribution of scores and the corresponding PCC for the center US-1 (global analysis). The reference used is shown in a side-by-side comparison with the center distribution.

**Figure 2:**
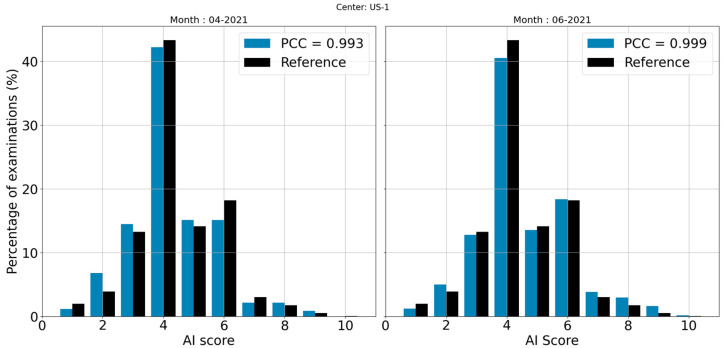
Distributions of scores corresponding to the local analysis of the months with the lowest PCC (**left**) and the highest PCC (**right**) for the center US-1; the corresponding reference is shown in a side-by-side comparison with each distribution.

The global analysis distribution of scores corresponding to the center US-1 and the reference used for comparison are displayed in Figure 1. The PCC is very close to 1 (0.998), and the graph confirms the similarity between the two distributions. Regarding local analysis, the months corresponding to the lowest and the highest PCCs (0.993 and 0.999) are displayed in Figure 2. For this example, the reference is superimposed on each of the distributions in order to facilitate the interpretation of the graphs. The distribution having the highest PCC value (0.999) is visually almost identical to the reference.

### 3.2. χ^2^ Test

χ^2^ test between each center distribution and its related reference yielded 0.85 for center US-1, 0.346 for US-2, 0.74 for US-3 and 0.78 for US-4. All the *p*-values are above the alpha significance threshold (0.05), which means that no significant difference between each center and its reference was found. The comparison between center US-1 and an unmatched reference led to a *p*-value of 0.008 (inferior to the alpha-value), thus showing a statistically significant difference between both distributions. Table 4 summarizes the results for the overall comparisons for each center.

### 3.3. Score-by-Score Difference

**Figure 3:**
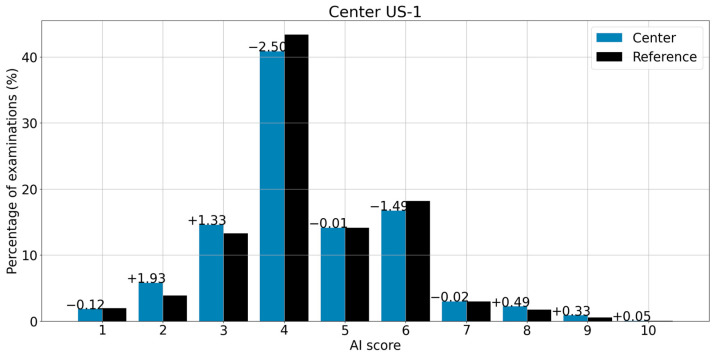
Distribution of scores for center US-1 and its corresponding reference (global analysis). The difference between every bin from the reference and the center appears above the top of each bin.

Figure 3 shows center US-1 and its reference distributions side by side to highlight the difference for each score (indicated above each pair of bins). The first pair of bins shows a decrease of 0.12 cases for score 1 compared to the reference, an increase of 1.93 cases for score 2 compared to the reference and so forth. Table 5 summarizes differences between the reference and centers US-2, US-3 and US-4, score by score.

### 3.4. Deviation Detection

**Figure 4:**
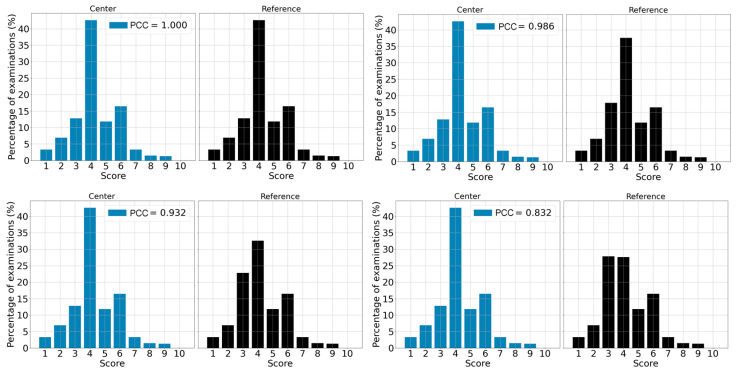
Example of 4 comparisons leading to 4 different PCC values. When the PCC is equal to 1, both distributions are identical. For the PCC value of 0.986, distributions are very similar. When the PCC is lower than 0.9, distributions are considerably different (for example when PCC is 0.832).

The PCCs between each center distribution and an unmatched reference were 0.374 for center US-1, 0.825 for US-2, 0.707 for US-3 and 0.364 for US-4. Regarding the χ² *p*-value, the estimates are 0.008 for center US-1, 0.32 for US-2, 0.22 for US-3 and 0.026 for US-4 as shown in Table 6. Knowing the upper limit of a deviation in terms of the PCC value requires defining a threshold. According to our data, 0.9 is the number that better defines a limit to separate deviations from normal behavior. To be more conservative, we chose a threshold of 0.95, which led to the following rule: if a given PCC is below 0.95, then the case is considered a deviation and further investigations are needed; if the PCC is higher than 0.95, then we can conclude that the system is behaving as expected. Figure 4 shows the effect of the difference between distributions on the resulting PCC.

### 3.5. Severity of a Shift

The severity of a shift was estimated using the Wasserstein distance (WD). When comparing two distributions consisting of scores between 1 and 10, the maximum W value is 9 – this is when we compare a distribution consisting of only 1 versus a distribution consisting of only 10. For subsequent calculations, we normalized the WD values by dividing by this maximum value.

**Figure 5:**
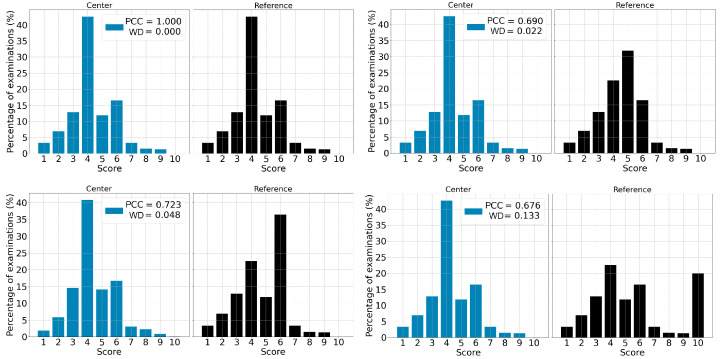
Example of four simulations where a shift takes place. The top left image represents two identical distributions resulting in a PCC of 1 and a WD of 0. The top right image represents a shift in the right distribution, where half of the population having a score of 4 now has a score of 5. The bottom left image represents a shift in the right distribution, where half of the population having a score of 4 now has a score of 6. The bottom right image represents a shift in the right distribution, where half of the population having a score of 4 now has a score of 10.

Figure 5 shows several simulated shifts where the PCC detects the deviation but fails to measure the severity of each shift, while WD is able to discriminate the level of severity.

**Figure 6:**
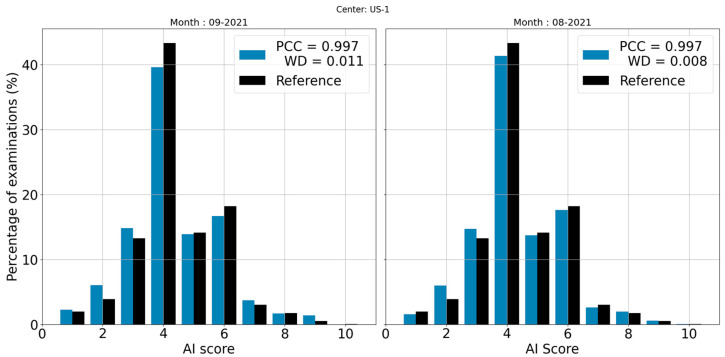
Score distributions from September (**left**) and August (**right**) 2021 for center US-1. In this example, both distributions have the same PCC value but have different WD values.

Figure 6 shows an example of two distributions where no deviation is detected. However, there is a distribution with a slightly higher shift than the other, resulting in a small difference in terms of WD value. Although visually the two distributions are almost identical and so are the PCCs, the WD values make it possible to identify the differences with greater precision. WD values can catch small differences and may be useful, especially in the case of a deviation where some shifts may have harmful consequences (such as deviations potentially leading to false negatives).

## 4. Discussion

This study aimed to propose a new methodology for monitoring an AI after its deployment in a clinical setting. Such monitoring is crucial since it indicates whether the predictions deriving from the AI concerning the patient’s health are as expected.

Feng et al. described several methods for monitoring clinical AI algorithms [11]. The most common tool described in their study is a control chart to display limits indicating the normal range of values for a given statistic. When the control limits of the chart are exceeded, an alarm is triggered, thus prompting human action (such as investigating the root cause of the deviation) [22]. The principle of these charts is to define values not to be exceeded, and in most applications the ground truth value is temporally close to the predicted one, which provides a quick a reference value for monitoring. These conditions make these charts unsuitable in the field of breast cancer detection [23,24].

Our proposed approach focuses on the comparison between the distribution of AI performance and a well-known and controlled distribution; this has not been investigated in other studies as far as we know.

Several metrics exist to estimate the degree of resemblance between two histograms. The most commonly used are bin-to-bin comparisons, which study only the corresponding bins of two histograms. The most commonly used bin-to-bin methods include the histogram intersection (HI), the Bhattacharyya distance (BD), the Kullback–Leibler divergence (KL), the histogram correlation (HC) and chi-squared (χ ²) statistics [25]. Swain and Ballard [26] proposed the HI metrics. The result of this calculation is the number of samples of the first histogram that have corresponding samples in the second histogram. The final score is between 0 and 1, the highest value being the most similar distribution. BD is a divergence measure yielding a geometric interpretation as the cosine of the angle between the distributions [27]. KL measures the differences between the two histograms from the information theory point of view, yielding the relative entropy of one histogram with respect to the other [28,29,30]. While this may be the most commonly used technique for comparing distributions, it has the disadvantage of being asymmetrical. HC indicates the degree of relationship between two variables with a single coefficient and it is directly derived from the Pearson’s correlation, thus measuring not just the strength but also the direction of a linear relationship between two variables [15]. This coefficient ranges between −1 (maximum negative relationship) and +1 (maximum positive relationship). Finally, the χ ² distance compares discrete probability distributions. It expresses the fit between observed and expected frequencies [31]. These different metrics provide equivalent information on the similarity of two distributions [25]. The main disadvantage of regarding the overlap (distance or intersection) between two histograms is that it does not consider the similarity of the non-overlapping part of the two distributions. The HC metric is able to consider both overlap and non-overlap parts by including bin variance in the calculation, thus better describing the resemblance of the behaviors of the two distributions. For this reason, our choice fell to the HC method.

The results of this study showed that the performance data collected in the different centers are very close to the desired values. Indeed, correlation values found in the present study, including the minimum and maximum value for all centers, were between 0.908 and 0.999, thus showing that the AI behaved as expected. Not a single significant difference was found when using the appropriate reference for comparison, since χ² tests *p*-values ranked from 0.346 to 0.853. The low PCC value found when an unmatched manufacturer is used for the reference shows that finding a difference could be the source of an alert, which would facilitate the detection of software malfunctions or deviations (such as an unsupported manufacturer or a version of the tool that is not suitable for the analyzed mammogram). The robustness of such a study comes from the large number of reports used for the statistical tests; this allows physicians to avoid hasty conclusions, which could be drawn from the immediate analysis of just a few cases [8].

The study has some known limitations. The set of reports used to define reference behavior is geographically limited to one region of the US, thus describing that specific population at that time. These data, compared to a population with different characteristics, can lead to a large and significant difference (even if there is not a specific problem concerning the AI behavior). An improvement could be to create references from more varied populations to have baselines more adapted to different centers around the world. Another point that has not been sufficiently explored here and may be another axis of improvement is to determine the alert level according to the difference between the reference and the center. Indeed, some results could raise low-level alerts, which would be informative but would not require any intervention, while other results could have more significant repercussions to ensure patient safety, such as reinforcing tests to eliminate any possibility of tool failure. In terms of implementing alerts, it then would be desirable to trigger a notification to users to inform them about the problem in a timely manner. The user could then have the choice to request an intervention from the AI designer.

It would also be interesting, in the context of long-term monitoring, to provide dashboards tracing all the drifts or deviations over a given time interval. This is so that the user can compare these results with the demographic data of his/her center and check whether these anomalies can be explained by phenomena such as a temporary increase in the average age or a change in the distribution of ethnic origins of the patients.

## 5. Conclusions

The results as well as the areas for improvement of the monitoring methodology described in this study are encouraging and worth development and integration in future AI versions. The more regular and efficient the monitoring, the more we can ensure the safety of decisions concerning the health of patients. Furthermore, this type of methodology is not limited to the intended use in the present study but can be extended to the evaluation of any software producing categorical predictions or scores, provided that a benchmark behavior is known.

Future research in this field includes extracting data to continuously check that the AI tool works as expected for long periods. This will include varying the thresholds of the used metrics to refine the alerts potentially generated.

## Figures and Tables

**Table 1:** Characteristics of the selected dataset.

Center	Manufacturer	Reports	AI-Version	Modality	Dates (Months)
US-1	Hologic^®^	18,470 (51 %)	1.2	FFDM	April 2021–February 2022 (11)
US-2	Hologic^®^	6227 (17 %)	1.3	FFDM	October 2021–March 2022 (6)
US-3	Hologic^®^	11,100 (30 %)	2.0.1	DBT	October 2021–October 2022 (13)
US-4	Hologic^®^	784 (2 %)	1.2	FFDM	July 2021–March 2022 (9)

**Table 2:** Characteristics of the selected reference dataset.

Number of Exams	AI Version Assessed	Manufacturer	Modality	Dates
13,433 (25.4%)	1.2	Hologic^®^	FFDM	December 2006–July 2019
25,330 (47.8%)	1.3	Hologic^®^	FFDM	October 2006–July 2019
14,187 (26.8%)	2.0	Hologic^®^	DBT	October 2006–July 2019

**Table 3:** PCC mean and standard deviation for each center.

Center	PCC Mean	PCC Standard Deviation
US-1	0.996	0.002
US-2	0.968	0.023
US-3	0.985	0.084
US-4	0.971	0.083

**Table 4:** Global PCCs and χ² *p*-values for each center.

Center.	Manufacturer and Modality	Version	PCC	χ² *p*-Value	Reference
US-1	Hologic FFDM	1.2	0.998 [min: 0.993, max: 0.999]	0.853	Hologic (FFDM), V 1.2
US-2	Hologic FFDM	1.3	0.975 [min: 0.923, max: 0.986]	0.616	Hologic (FFDM), V 1.3
US-3	Hologic DBT	2.0	0.995 [min: 0.972, max: 0.998]	0.743	Hologic (DBT), V 2.0
US-4	Hologic FFDM	1.2	0.994 [min: 0.962, max: 0.982]	0.785	Hologic (FFDM), V 1.2

**Table 5:** Difference between each center and the reference, score by score.

Center	AI Score									
	1	2	3	4	5	6	7	8	9	10
US-2	+0.37	−4.8	+5.54	+5.82	−3.71	−5.71	+0.41	+1.29	+0.65	+0.14
US-3	+0.94	−1.64	−2.21	−0.95	−0.11	+1.22	+0.76	+1.18	+0.7	+0.11
US-4	+1.47	+3.09	−0.44	−1.02	−2.25	−1.79	+0.3	−0.18	+0.82	+0

**Table 6:** Global PCCs and χ² *p*-values for each center, when compared to a wrong reference.

Center	Manufacturer and Modality	Version	PCC	χ² *p*-Value	Reference	Mismatch Cause
US-1	Hologic FFDM	1.2	0.714	0.026	Hologic (DBT), V 2.0	Wrong modality, Wrong version
US-2	Hologic FFDM	1.3	0.825	0.320	Fuji (FFDM), V 1.3	Wrong manufacturer
US-3	Hologic DBT	2.0	0.707	0.221	Fuji (FFDM), V 1.3	Wrong manufacturer, Wrong version
US-4	Hologic FFDM	1.2	0.364	0.026	Fuji (FFDM), V 1.3	Wrong manufacturer, Wrong version

## Data Availability

No new data were created or analyzed in this study. Data sharing is not applicable to this article.
